# Catastrophic storms, forest disturbance, and the natural history of Swainson's warbler

**DOI:** 10.1002/ece3.11099

**Published:** 2024-03-13

**Authors:** Gary R. Graves

**Affiliations:** ^1^ Department of Vertebrate Zoology MRC‐116, National Museum of Natural History, Smithsonian Institution Washington DC USA; ^2^ Center for Macroecology, Evolution and Climate, Globe Institute University of Copenhagen Copenhagen Ø Denmark

**Keywords:** hurricanes, *Limnothlypis swainsonii*, Swainson's warbler, tornadoes

## Abstract

The core breeding range of Swainson's warbler (*Limnothlypis swainsonii*) overlaps a zone of exceptionally high tornado frequency in southeastern North America. The importance of tornadoes in creating breeding habitat for this globally rare warbler and other disturbance‐dependent species has been largely overlooked. This paper estimates tornado frequency (1950–2021) and forest disturbance in the 240 counties and parishes in which breeding was documented from 1988 to 2014. The frequency of destructive tornadoes (EF1‐EF5) varied 6‐fold across the breeding range with a peak in the Gulf Coast states. Counties from east Texas to Alabama experienced the lowest median return interval of 5.4 years per 1000 km^2^, resulting in approximately 2477 ha of forest damage per 1000 km^2^ per century, based on current forestland cover. Tornadoes were significantly less frequent north and east of the core breeding range, with return intervals increasing to 9.1 years per 1000 km^2^ for breeding counties on the Atlantic coastal plain, 10.2 years per 1000 km^2^ in the Ozark Mountains, and 32.3 years per 1000 km^2^ in the Appalachian Mountains. Breeding counties within 150 km of the coastline from east Texas to North Carolina are also subjected to the highest frequency of hurricanes in the Western Hemisphere. Hurricanes often cause massive forest damage but archived meteorological and forestry data are insufficient to estimate the aggregate extent of forest disturbance in breeding counties. Nevertheless, the combined impact of tornadoes and hurricanes in the pre‐Anthropogenic era was likely sufficient to produce a dynamic mosaic of early‐successional forest crucial for the breeding ecology of Swainson's warbler. To ensure the long‐term survival of this rare warbler, it is advisable to develop habitat management plans that incorporate remote sensing data on early‐successional forest generated by catastrophic storms as well as anthropogenic activities.

## Graphical abstract

Swainson's warbler is a rare disturbance‐dependent songbird that breeds in forests of southeastern United States. This paper explores the role of catastrophic storms in creating breeding habitat in its contemporary breeding range.

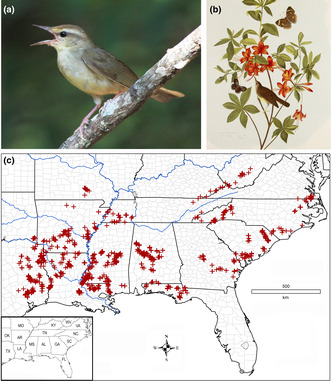

## 1 INTRODUCTION

The serendipitous 1833 discovery of Swainson's warbler (*Limnothlypis swainsonii*) (Audubon, 1834) and Bachman's warbler (*Vermivora bachmanii*) (Audubon, 1833) by the Reverend John Bachman, near Parkers Ferry, South Carolina, sparked a question that has long perplexed ornithologists. Why are they so rare? These enigmatic southern warblers are linked by history, rarity, and geography. Their habitat preferences were apparently similar, with active nests of the two species discovered within 10 feet (3 m) of one another (Wayne, 1907). Bachman's warbler seemingly reached a population peak in the 1880s and 1890s when dozens were observed during spring migration in coastal Louisiana (Galbraith, 1888) and northern Florida (Brewster, 1891). However, this diminutive warbler was always regarded as a rare and locally‐distributed species and relatively little was discovered about its natural history while breeding individuals were still observable (Hamel, 1986; Stevenson, 1972). After a century of unexplained decline, it likely became extinct in the 1960s (Elphick et al., 2010), and was officially declared extinct in 2023 (U. S. Fish and Wildlife Service, 2023).

The extinction of Bachman's warbler was perhaps best explained by Terborgh (1989), who pointed out that extensive deforestation on its Cuban wintering grounds, coinciding with the 19th‐ and 20th‐ century‐expansion of the sugar industry, drastically reduced the carrying capacity during the nonbreeding season. The diminished population of males and females faced the insurmountable challenge of finding one another in the vastly larger breeding range that extended from the lower Mississippi Valley to coastal South Carolina. This problem was magnified by large‐scale logging of old‐growth bottomland forest following the Civil War through the 1930s (Williams, 1989). Mass cutting resulted in millions of hectares of early‐successional woodland and an overwhelming abundance of optimal breeding sites for yearlings, which typically settle many kilometers from their natal locations. Once the global population declined to a critical tipping point, natal dispersal quickly drove the species to extinction. The last few males observed in the 1950s and 1960s sang persistently but failed to attract mates (Terborgh, 1989).

**FIGURE 1:**
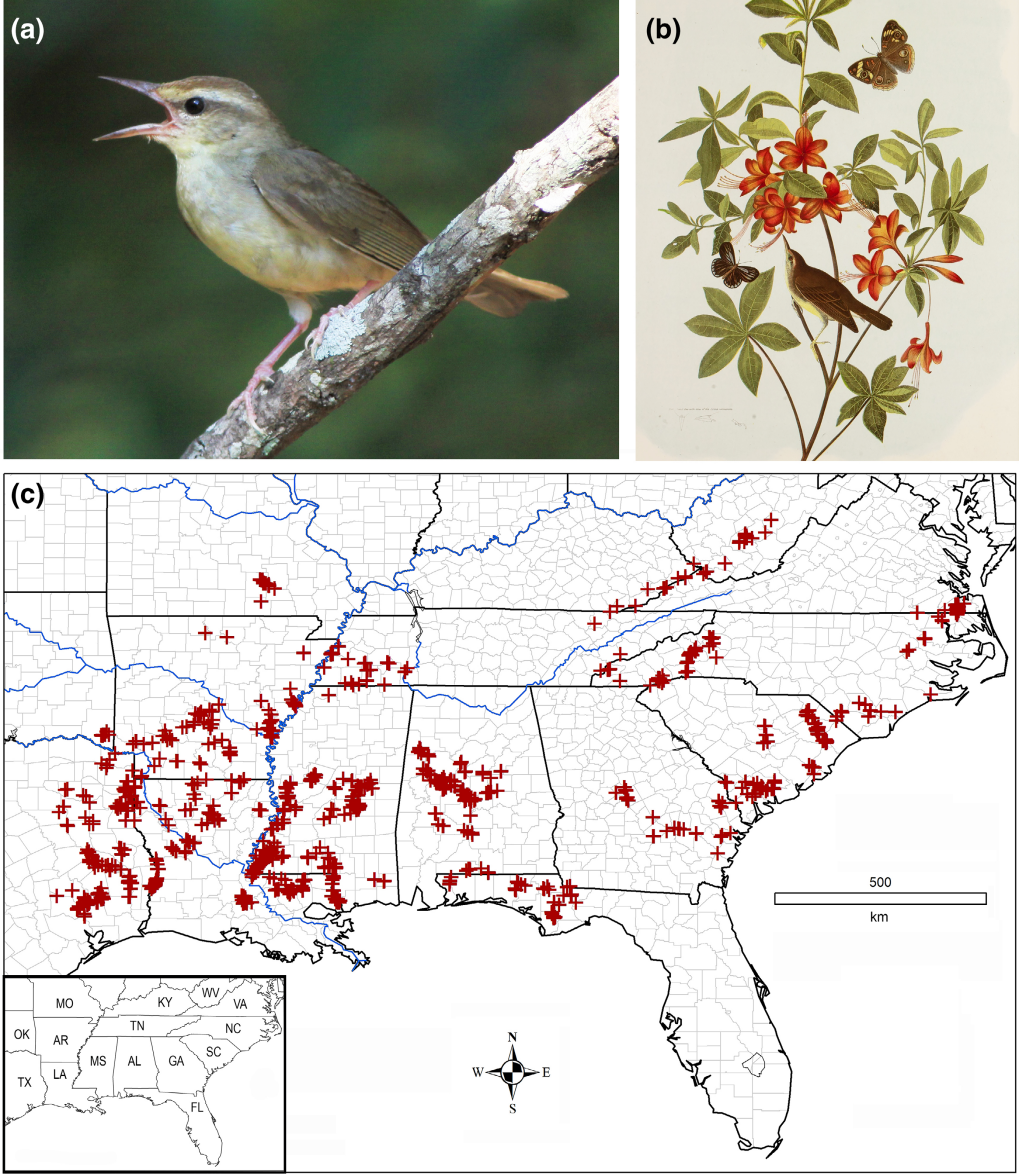
Swainson's warbler (*Limnothlypis swainsonii*). (a) Singing male (Red River Parish, Louisiana), (b) Lithograph of painting by John James Audubon (1834) of the type specimen collected by John Bachman in 1833. This specimen (USNM 2901) is deposited in the National Museum of Natural History, Smithsonian Institution, (c) Geolocated breeding territories in 240 counties and parishes from 1988 to 2014. Major rivers are highlighted in blue, county borders (parishes in Louisiana) in pale gray, and state boundaries in black. Inset map: AL (Alabama), AR (Arkansas), FL (Florida), GA (Georgia), KY (Kentucky), LA (Louisiana), MO (Missouri), MS (Mississippi), NC (North Carolina), OK (Oklahoma), SC (South Carolina), TN (Tennessee), TX (Texas), VA (Virginia), and WV (West Virginia).

Swainson's warbler could well face a similar fate in the coming century. This secretive species, which is nowhere common, now holds the unfortunate distinction of being the rarest breeding songbird on the mainland of southeastern North America (Figure 1). The sparsely distributed breeding population, recently estimated at 156,260 (Partners in Flight, 2022; Will et al., 2020), occupies a fragmented range that encompasses 1.14 million km^2^ from Texas to West Virginia (Partners in Flight Science Committee, 2013). The disappearance of peripheral populations in Delaware, Maryland, Missouri, and Illinois in recent decades signals a retraction toward the core breeding range in the Gulf Coast states. Population size is believed to be limited by deforestation on the breeding grounds (Graves, 2001, 2002) and wintering range in the Caribbean basin (Terborgh, 1989), in concert with changes in forest management policy (Graves & Tedford, 2016), flooding (Graves, 2001, 2002; Meanley, 1971; Reiley et al., 2013), and cowbird brood parasitism (Benson et al., 2010; Meanley, 1969).

Following its discovery, Swainson's warbler was virtually lost to science for 50 years until breeding sites were found in South Carolina and Georgia in the 1880s (Brewster, 1885). The resulting flurry of natural history notes focused on descriptions of nests and eggs, nest microhabitat, and geographic range extensions (Graves et al., 1996; Meanley, 1971), but contained little substantive information on landscape characteristics or the degree of natural and anthropogenic disturbance on breeding territories. The sole 19th century account from unequivocal old‐growth forest was documented in southeastern Missouri where Widmann (1895) found the warbler to be an uncommon breeding bird in bottomland forest where cypress and sweet gun towered over a mid‐story of hornbeam, ash and dogwood. The profusion of vine tangles and thickets of pawpaw, hazel, spicebush, and Hercules club at the site are indicative of disturbance and substantial light penetration to the understory. Regrettably, by the time the first quantitative habitat analysis was conducted by Eddleman et al. (1980), the opportunity to study the species in such primeval settings had largely passed. Consequently, our understanding of Swainson's warbler habitat selection is based mostly on studies in anthropogenically‐altered landscapes.

Contemporary populations of Swainson's warbler breed mostly in regenerating shelterwood cuts and clearcuts on commercial forestry lands where they reach their peak abundance. Smaller numbers also breed in a baffling variety of secondary habitat types (Bassett‐Touchell & Stouffer, 2006; Brooks & Legg, 1942; Graves, 2002, 2015, 2019, 2020; Henry, 2004; McNair, 2019; Meanley, 1971; Wood, 2014) that share a common characteristic—natural or anthropogenic disturbance, or a physiognomy that resembles the regenerative outcome of disturbance. Breeding territories invariably exhibit a high density of understory stems and/or foliage (Graves, 2002, 2015; Graves & Tedford, 2016) in patches sufficiently spacious to accommodate the warbler's expansive territories (Anich et al., 2009; Graves, 2001), which averaged 6.5 ha In the most intensively studied population in the lower Mississippi Valley. Habitat suitability is also influenced by flooding (Graves, 2001; Reiley, 2012), soil type, and soil moisture levels (Graves, 2002, 2015). These factors affect the deposition and decay of leaf litter and abundance and diversity of terrestrial litter arthropods (Brown, Benson, & Bednarz, 2011; Savage et al., 2010), which are essential resources for this dead‐leaf foraging specialist (Graves, 1998). The entire breeding distribution is characterized by moderate annual precipitation, and only a few peripheral breeding sites in Texas lie outside the 1000 mm‐isohyet.

**FIGURE 2:**
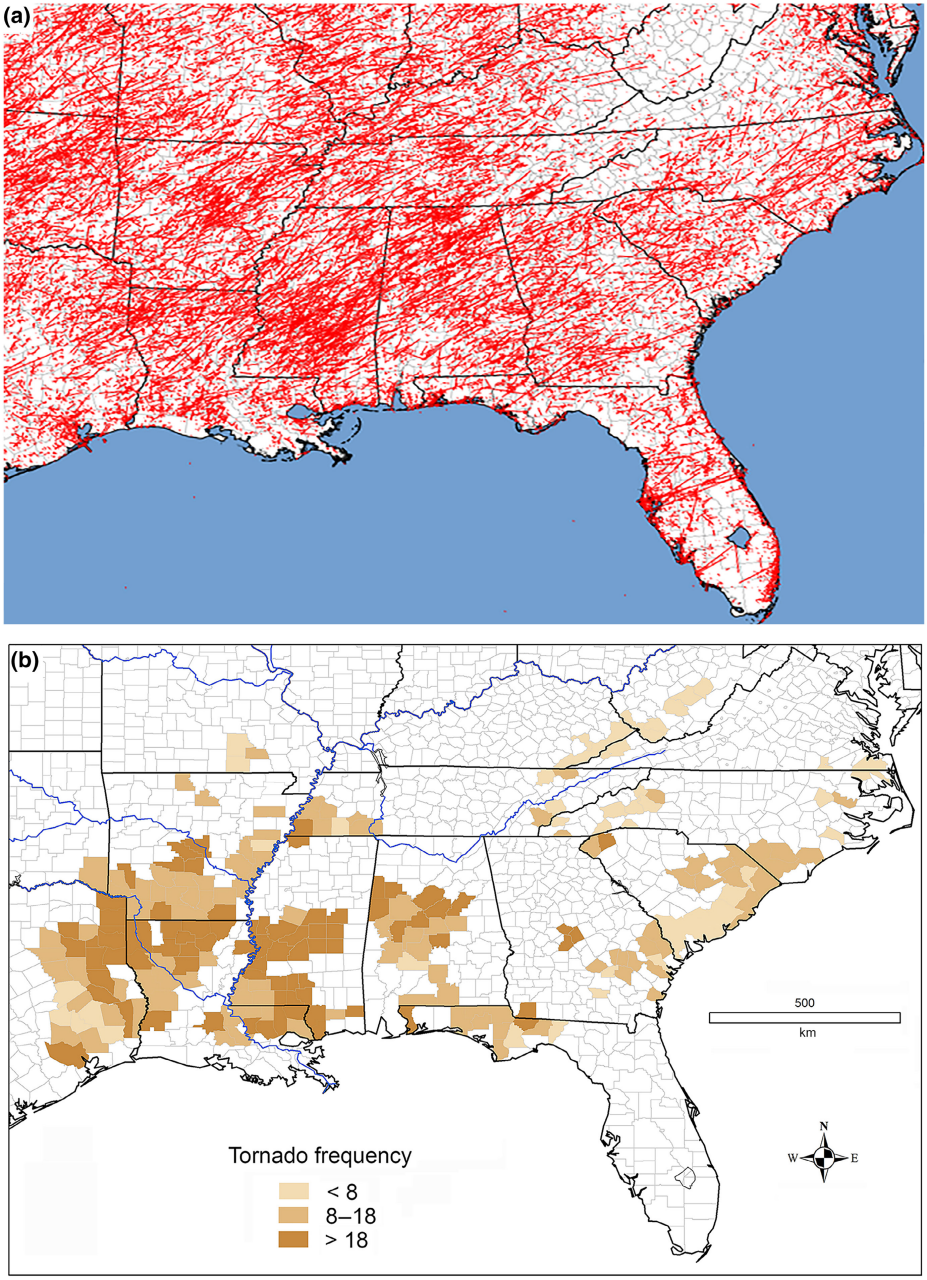
Tornadoes in southeastern United States. (a) Tornado tracks (EF0‐EF5) in southeastern United States (1950–2021). Modified from https://www.spc.noaa.gov/gis/svrgis/images/tornado. (b) Tornado frequency (EF1–EF5; per 1000 km^2^ per century) in counties with documented breeding territories of Swainson's warbler.

A zone of exceptional tornado activity in the Gulf Coast states (Brooks et al., 2003; Gensini & Brooks, 2018), known as “Dixie Alley” among meteorologists (Dixon et al., 2011; Frazier et al., 2019; Gagan et al., 2010), extends from east Texas to Alabama. More than 75% of the global population of Swainson's warbler breeds in this geographic belt (Figure 2, Table 1). It is unclear whether this spatial overlap is coincidental or causative but field researchers are well aware of the warbler's affinity for disturbance gaps created by catastrophic storms. Despite this awareness, none of the 16 quantitative studies of breeding habitat has addressed the role of catastrophic storms in the formation of optimal breeding habitat (Bassett‐Touchell & Stouffer, 2006; Bednarz et al., 2005; Benson, 2008; Brown et al., 2009; Chartier, 2014; Eddleman et al., 1980; Graves, 2001, 2002; Graves & Tedford, 2016; Henry, 2004; Peters et al., 2005; Reiley et al., 2013; Somershoe et al., 2003; Thomas et al., 1996; Thompson, 2005; Wright, 2002). The omission stems in part from the limited size of study plots and the modest spatial scales of conservation programs. More significantly, it reflects the prevalence of anthropogenic disturbance and commercial forestry, which have supplanted natural events as the primary generators of early‐successional forest within the warbler's breeding range (Graves, 2015).

**TABLE 1:** Breeding population estimates for Swainson's warbler at the state level derived from *Population Estimates Database, version 3.1* (Partners in Flight, 2022).

Alabama	45,370
Texas	31,100
Louisiana	16,100
Arkansas	13,000
Mississippi	13,000
North Carolina	9985
South Carolina	6700
Georgia	6340
Florida	5200
Tennessee	2775
Virginia	2490
West Virginia	2400
Kentucky	1800
Total	156,260

*Note*: Breeding populations in Oklahoma and Missouri were too small to warrant their inclusion.

**FIGURE 3:**
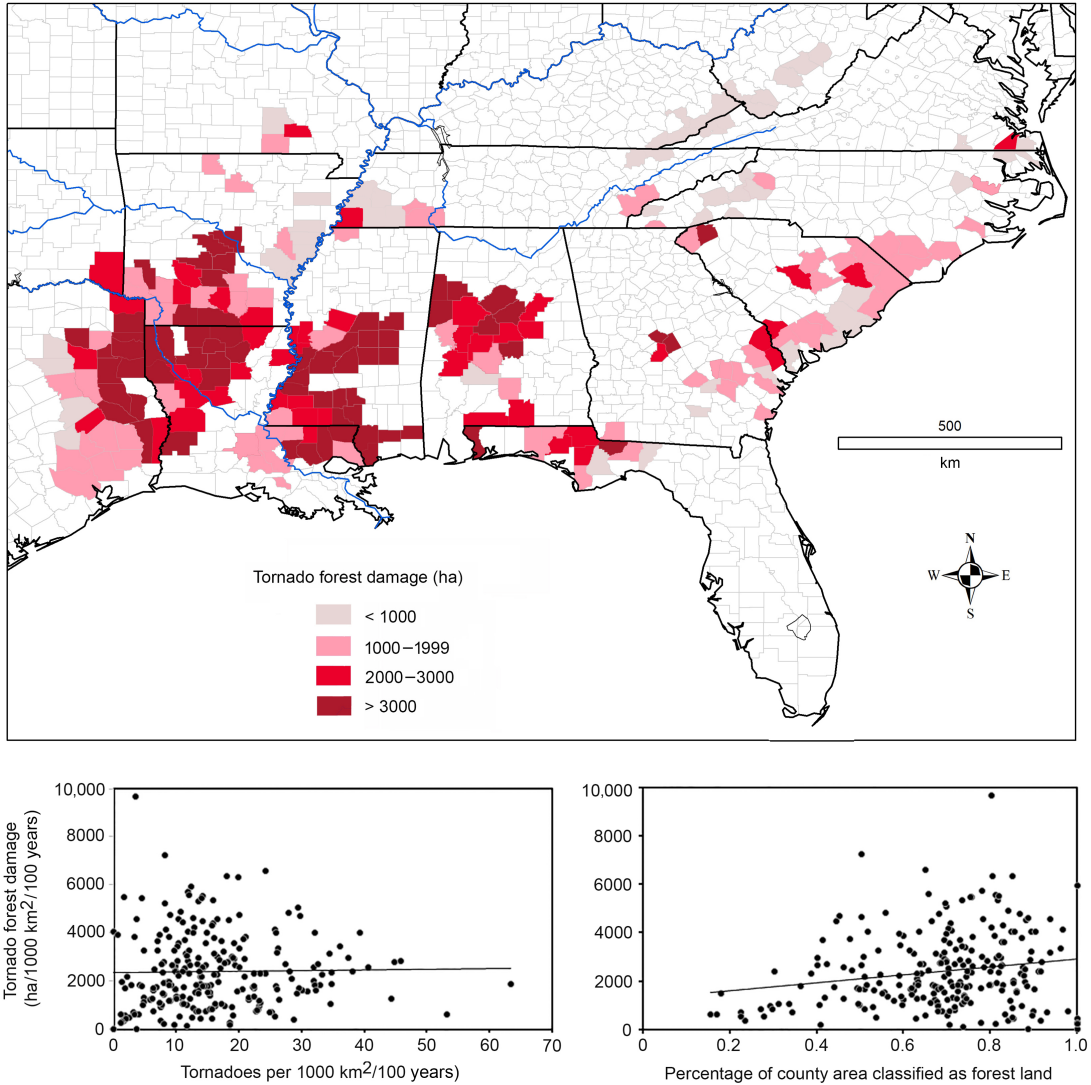
Tornado forest damage (ha per 1000 km^2^ per century). Estimates of county‐level damage were uncorrelated with area‐adjusted tornado frequency, because of extensive deforestation in some counties with high tornado frequencies (*R*_s_ = 0.10, *p* = .11). Estimated forest damage exhibited a weak correlation with the percentage of forest cover despite the wide scatter of data points (*R*_s_ = 0.16, *p* = .014).

This study characterizes the spatial gradients of tornado frequency in the southeastern United States and estimates tornado forest disturbance in context to the breeding ecology of Swainson's warbler. The analyses relied on three primary data sources. A geolocated archive of breeding territories (*n* = 1717) located in 240 counties and parishes across 15 states served as the geographic template for storm data analysis. Tornado data for these areas were obtained from NOAA's National Centers for Environmental Information (National Centers for Environmental Information, 2022a, 2022b). Additionally, county‐level data for forestland cover were acquired from the Forest Inventory and Analysis (FIA) database maintained by the USDA Forest Service (USDA Forest Service, 2022). These datasets facilitated the estimation of tornado frequency (Figure 2) and the extent of tornado forest disturbance in the contemporary breeding range (Figure 3).

**FIGURE 4:**
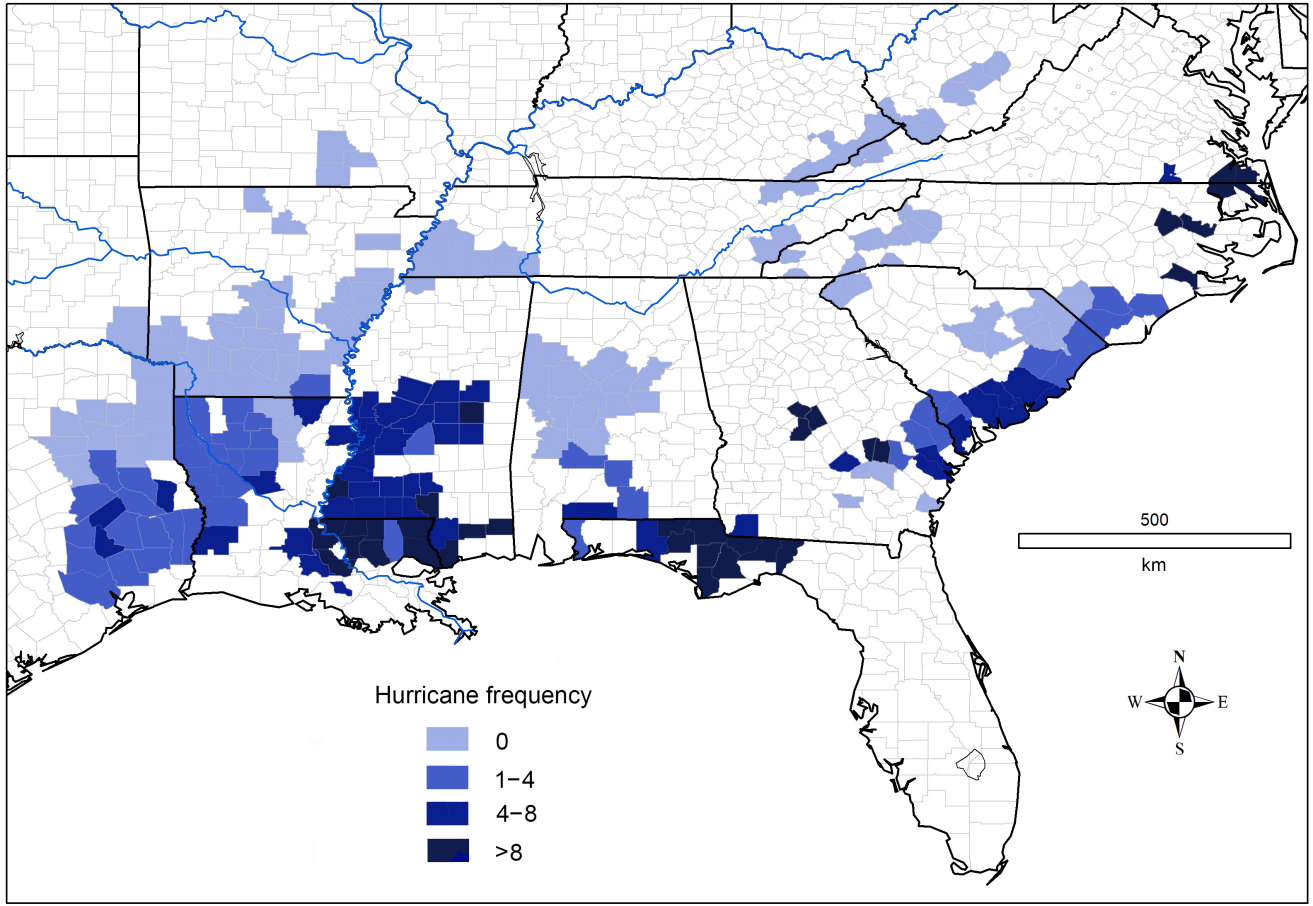
Hurricane frequency (per 1000 km^2^ per century) in Swainson's warbler counties.

A parallel analysis was conducted for hurricanes in southeastern United States (National Centers for Environmental Information, 2022a). Although the coastal‐to‐inland gradient in hurricane frequency is quite clear (Figure 4), county‐level estimates of hurricane forest damage could not be determined owing to data insufficiency. Both tornadoes and hurricanes are reviewed here despite their significant differences in scale and destructive potential. The resulting analyses provide valuable insight into the regional gradients of catastrophic storms and forest damage within the breeding range of Swainson's warbler. This information may be crucial for the effective management of this species and other disturbance‐dependent songbirds in the southeastern United States.

## 2 METHODS

### 2.1 Geolocation of territorial warblers

From 1988 to 2014, I surveyed breeding populations of Swainson's warbler in 15 states as part of a comprehensive study of the warbler's natural history (Graves, 1998, 2001, 2002, 2015, 2020; Graves & Tedford, 2016). These surveys targeted Swainson's warbler and were not incidental components of broader community censuses. Territorial males were documented in 240 counties and parishes by song recordings (see *Data availability*). I use the terms “warbler counties” and “breeding counties” interchangeably to refer to these administrative jurisdictions. Surveys were conducted during the breeding period, which began on 22 April in the Gulf Coast states and ended on 30 June in the Appalachian Mountains. I surveyed a wide spectrum of forestland and shrubland habitats, broadly classified as “forest land” by the USDA on public and private land and along waterways. Habitat physiognomy was sampled quantitatively on 7.2% of breeding territories, but these data were geographically clustered at only eight of hundreds of field sites (Graves, 2001, 2002; Graves & Tedford, 2016). Most breeding territories of this monogamous species were located using playback of songs, utilizing a protocol that was field‐tested and fine‐tuned in the late 1980s (Graves, 1996, 2001). Territorial males respond to playback by approaching the song source and delivering agitated “chip” notes, but usually refrain from singing until the playback source retreats or playback ends. Response to playback, mate‐guarding, persistence during “playback‐and‐follow” trials, and counter‐singing with other males were regarded as evidence of territoriality. Mist‐netting or other handling was not required to document territoriality. The geographic coordinates of territories were recorded on site with Garmin™ GPS receivers (post‐1998) or with Google Earth Pro from field notes and maps. A significant strength of this fieldwork archive is that it was compiled by a single senior investigator using comparable methods for 26 years. Data from other potential sources, such as eBird (https://ebird.org), were not used because of observer competency issues, geographic imprecision of checklists, the absence of photographic or auditory documentation, uncertainties about breeding status, and the widespread confusion between song variants of Swainson's warbler and Louisiana waterthrush (*Parkesia motacilla*).

### 2.2 Tornadoes: Overview and data

Tornado data spanning 71 years (1 January 1950 to 6 February 2021) for the 240 warbler counties were obtained from the NOAA database (National Centers for Environmental Information, 2022a). Tornado intensity was measured on the Fujita (F) scale until 2007 when the National Weather Service adopted the Enhanced Fujita (EF) scale (Doswell et al., 2009; Potter, 2007). The F and EF scales have six intensity categories indexed on damage to human structures and softwood and hardwood trees. Intensity scales are proxies for wind speed, which range from EFO (105–137 km h^−1^ for 3‐s gusts) to EF5 (>320 km h^−1^) (Edwards et al., 2013). Minimum wind speeds are slightly lower on the F scale than the EF scale for each category, e.g., F1 (126 km h^−1^) versus EF1 (137 km h^−1^). The frequency distribution of tornadoes by EF intensity scale appears to follow a power law in which the number of EF2 tornados is approximately half the number of EF1 tornadoes and so on (Elsner, Jagger, Widen, & Chavas, 2014). While low intensity tornadoes (F0 and EFO) are capable of stripping foliage and breaking small branches, they seldom result in windthrow or canopy damage that is visible by remote sensing (Zenoble & Peterson, 2017). EF1 (F1) tornadoes can uproot or topple mature trees, while EF2 (F2) and EF3 (F3) events can snap them off. High‐end EF3 (F3) tornadoes can debark trees, leaving only stubs of largest branches remaining on standing trunks. EF4 (F4) and EF5 (F5) tornadoes can cause catastrophic damage and are capable of reducing mature forests to barren debris fields.

The contiguous United States has experienced an average of 939 tornadoes per year (with a median of 915) from 1950 through 2021 (National Centers for Environmental Information, 2022b). The majority occurred east of the Rocky Mountains. Brooks et al. reported 495 tornadoes per year with an intensity rating of EF1 or higher from 1954 to 2013 (Brooks et al., 2014). In this study, I limited the analysis to intense tornadoes (>EF1 or F1) capable of creating large disturbance gaps in mature forests. An important limitation of the NOAA database is that it likely undercounts less severe tornadoes (EFO and EF1) in sparsely populated counties (Anderson et al., 2007; Potvin et al., 2022). Consequently, the analysis underestimates the actual frequency of tornadoes in southeastern United States. This study did not include data on derechos, downbursts, and microbursts, as county‐level records for these events were either unavailable or incomplete in the NOAA database. Although derechos are rarer than tornadoes, they can cause catastrophic forest disturbance equivalent to that generated by major tornadoes and hurricanes (Ashley & Mote, 2005; Bentley & Mote, 1998; Peterson, 2000).

Tornadoes in the NOAA database are tabulated by county. Average county area varies considerably among states: e.g., Alabama ($\bar{x}$ = 2030 km^2^) versus Georgia ($\bar{x}$ = 968 km^2^). Larger counties tend to experience more tornadoes than smaller counties, all else being equal (*n* = 240; *R*_s_ = 0.58, *p* < .0001). The most intense tornadoes often cross county lines (Burow et al., 2020; Cannon et al., 2016), which amplifies spatial autocorrelation in county‐level counts of tornado occurrence. To mitigate the effects of county size, I standardized the tornado return frequency and return interval by county area (per 1000 km^2^ per century).

Smoothed, interpolated maps of tornado frequency in the continental United States, which include less intense tornados (EF0) over significantly shorter time intervals, are available in the meteorological literature (Brooks et al., 2003; Dixon et al., 2011; Frazier et al., 2019; Gensini & Brooks, 2018). However, using simple area‐standardized frequencies at the county level, as presented in this paper, offers transparency in both methodology and graphic output. This approach does not hinge on grid cell size, kernel density, or smoothing algorithms, providing a more straightforward and clear representation of tornado frequency in warbler counties.

### 2.3 Tornado track metrics

Tornado track length is defined as the distance traveled by tornadoes on the ground, while track width is the maximum‐recorded width of the tornado track. Median track length of tornadoes reported for the conterminous United States from 2007 to 2013 (Elsner, Jagger, & Elsner, 2014) increased monotonically with tornado intensity: EF1 (4.4 km, *n =* 2642), EF2 (10.0 km, *n =* 818), EF3 (23.3 km, *n* = 232), EF4 (34.8 km, *n =* 57), and EF5 (59.0 km, *n =* 9). The 1925 Tri‐State tornado, which started in Missouri and ended in Indiana (Johns et al., 2013), had a continuous track of 243 km (378 km including gaps). Median track width varies with tornado intensity: EF1 (91 m), EF2 (229 m), EF3 (549 m), EF4 (805 m), and EF5 (1920 m) (Elsner, Jagger, & Elsner, 2014). The 2013 El Reno tornado in Oklahoma holds the record for a maximum track width of 4.2 km (Bluestein et al., 2015). The ratio of median track length to width decreases with tornado intensity (EF1 = 48.0, EF2 = 43.9, EF3 = 42.4, EF4 = 43.3, and EF5 = 30.7).

I used several simplifying assumptions to estimate the areal footprint of forest disturbance. Assuming a rectangular track, the disturbance footprint can be roughly estimated by multiplying median track length by median track width (Cannon et al., 2016; Fujita, 1971). The width of individual tornado tracks often varies (Cannon et al., 2016; Karstens et al., 2013; Zenoble & Peterson, 2017), with patches of severe forest damage nested within less severely damaged patches that dissolve away from the center (Cannon et al., 2016). The degree of forest damage within the footprint will vary, from broken branches and stripped foliage at the track margins to toppled and snapped trees near the center. Tornado tracks often exhibit gaps (zero track width) with no severe forest damage. A study of 50 tornado tracks revealed a mean gap length of 13% of total track length (Zenoble & Peterson, 2017). I used this percentage as a correction factor in estimates of median footprint area for tornadoes: EF1 (35 ha), EF2 (199 ha), EF3 (1115 ha), EF4 (2439 ha), and EF5 events (9850 ha) (metadata from Elsner, Jagger, & Elsner, 2014). The mean frequency‐weighted footprint for all tornadoes (197 ha) was used to estimate forest disturbance gradients.

### 2.4 Forestland cover and tornado disturbance

Forest ecologists have conducted detailed studies on the impact of tornadoes on forest structure in eastern North America (Everham & Brokaw, 1996; Greenberg & Collins, 2016). These have focused on windthrow patterns produced by case events (Cannon et al., 2016; Peterson & Pickett, 1995), canopy structure and light penetration (Willson et al., 2020), and the resiliency of tree species to high winds (Peterson, 2007; Shirakura et al., 2006). My study aimed to provide a general approximation of historical rates of tornado forest damage in the 240 warbler counties by combining county‐level data for tornado records and forestland cover.

County‐level data for “forest land” coverage were obtained from the 2015 Forest Inventory and Analysis (FIA) database (USDA Forest Service, 2022). This database provides a coarse temporal snapshot of nominal forestland cover in the 240 warbler counties. Forestland cover varied widely, from 16 to 100% among the breeding counties, with nearly 6% of counties having >75% of land area classified as nonforest and more than 65% of counties having >25% of land area similarly classified. I used the median tornado footprint (197 ha), the area‐adjusted tornado frequency (tornadoes per 1000 km^2^ per century), and forestland coverage (%) to calculate rates of tornado forest damage (hectares per 1000 km^2^ per century) in the 240 warbler counties (Figure 3).

### 2.5 Hurricanes: Overview and data

County‐level data for hurricanes from 1 January 1996 to 1 April 2021 (25 years) were obtained from the NOAA National Centers for Environmental Information (National Centers for Environmental Information, 2022a). Hurricane intensity is scaled on the Saffir‐Simpson index ranging from Category 1 (wind speeds 119–153 km h^−1^) to Category 5 (wind speeds >252 km h^−1^) (https://www.nhc.noaa.gov/pdf/sshws.pdf). Category 1 hurricanes can snap large branches and topple shallowly rooted trees, while Category 2 storms can snap off mature trees. Major hurricanes (Category 3 or higher) may level mature forests. In general, economic damage to human infrastructure increases by a factor of four for every increase in category (Pielke et al., 2008) and forest disturbance may scale similarly with hurricane category. This study excluded tropical storms (<119 km h^−1^), although they can cause significant forest damage. Hurricanes at landfall are often >50 km in width (Irish et al., 2008), greater than the average widths of counties in the study area. However, because a hurricane may affect only a small portion of a county, I standardized hurricane incidence by county area (hurricanes per 1000 km^2^ per century; Figure 4), as was done for tornado data (Figure 2). County‐level estimates of hurricane forest damage could not be determined owing to the lack of county‐level data on the damage footprint of hurricane force winds.

### 2.6 Statistical analyses

County‐level tornado and hurricane frequencies were tested for goodness of fit to a normal distribution. The Lilliefors test showed significant deviation from normality for both tornado (*D* = 0.11, *p* = <.0001) and hurricane frequencies (*D* = 0.27, *p* < .0001), with right‐skewed and leptokurtic distributions. I therefore focused on median rather than mean values in statistical tests. Spearman rank correlation coefficients (*r*_s_) were used to evaluate the strength of correlations, while regional differences in tornado and hurricane frequency were evaluated with the Mann–Whitney *U*‐test. This test combines the distributions of two groups of values into a single sample and then assesses the range and location of the lowest group's distribution within the overall sample range against a ranked distribution that approaches normality (Hollander & Wolfe, 1973). Because one or both group sizes were larger than 20 in all comparisons, I used the normal approximation to calculate a *z*‐value for the Mann–Whitney *U‐*test. All *p‐*values are two‐tailed (*α* = 0.05). Statistical analyses were performed in R (version 4.2.0) with RStudio interface (version 2022.02.2) with the R Stats Package (RStudio Team, 2020).

## 3 RESULTS

### 3.1 Tornado return frequency

The 240 breeding counties examined in this study exhibited a median tornado frequency of 14 per 1000 km^2^ per century, with a range of 0 to 64 (Figure 2). Among the 145 breeding counties located within the meteorological “Dixie Alley,” which encompasses eastern Texas, southeastern Oklahoma, southern Arkansas, Louisiana, Mississippi, Alabama, and western Florida, and hosts the majority of breeding populations, the median tornado frequency was 18.5 tornadoes per 1000 km^2^ per century. Within this region, 127 of 145 counties (87.6%) reported a frequency of 10 or more tornadoes per 1000 km^2^ per century. The Ozark Mountains in Arkansas and Missouri, situated along the northern periphery of the breeding range, experienced a tornado frequency of 9.8 tornadoes per 1000 km^2^ per century, while counties in the Atlantic coastal plain reported a frequency of 11.0 tornadoes per 1000 km^2^ per century. The lowest tornado frequencies were observed in the Appalachian Mountains of West Virginia, Kentucky, Virginia, Tennessee, North Carolina, and South Carolina, with a median of 3.1 tornadoes per 1000 km^2^ per century.

### 3.2 Forest disturbance by tornadoes

Based on current percentages of forestland cover, tornado forest disturbance (Figure 3) in breeding counties was estimated to range from zero to 9656 ha per 1000 km^2^ per century (median = 2134 ha). These findings can also be interpreted as a forecast of future forest damage, assuming that there is no change in forestland area and if the frequency and intensity of tornadoes remain constant. Counties in the Gulf Coast states exhibited the highest rate of tornado forest damage (median = 2477 ha per 1000 km^2^ per century). This rate was significantly higher than estimates for the Atlantic coastal plain (median = 1461 ha; Mann–Whitney, *Z* = 6.27, *p <* .00001), Ozark Mountains (median = 1446 ha; Mann–Whitney, *Z* = 2.41, *p =* .016), and Appalachian Mountains (median = 470 ha; Mann–Whitney, *Z* = 7.28, *p* < .00001).

### 3.3 Hurricanes

The frequency of hurricanes in the 240 warbler counties ranged from zero to 40 per 1000 km^2^ per century, with a median of 1.6 per 1000 km^2^ per century. Hurricane force winds regularly extend 200 km inland along the Gulf coast, and occasionally to 400 km in the lower Mississippi Valley (Figure 4), but rarely more than 150 km inland on the Atlantic coast. The frequency of hurricanes in the Gulf Coast states (median = 3.9, *n* = 121 counties), including northwestern Florida, was similar to that observed on the Atlantic coastal plain (median = 3.3, *n* = 47; Mann–Whitney, *Z* = 0.25; *p* = .80). Overall, 28.8% (69 of 240) of counties experienced ≥5 hurricanes per 1000 km^2^ per century. The frequency of tornadoes and hurricanes was weakly correlated (*r*_s_ = 0.15; *p* < .02; *n* = 240). Many inland areas with high tornado frequencies, such as eastern Arkansas and western Tennessee, experienced no hurricane force winds, while some Atlantic coastal counties with high hurricane frequencies experienced few tornadoes. The greatest concentration of severe storms, combining tornadoes and hurricanes, occurred in a geographic belt from Texas to Alabama and northwestern Florida, with a band of exceptionally high frequency that extended from the eastern parishes of Louisiana northward through Mississippi. The Appalachian region stood out as having a relatively low frequency of tornadoes and no hurricane‐force winds.

## 4 DISCUSSION

The core breeding range of Swainson's warbler spatially coincides with the hemispheric peaks of tornado frequency (Brooks et al., 2003; Dixon et al., 2011; Frazier et al., 2019; Gensini & Brooks, 2018) and hurricane incidence (Chambers et al., 2007; Doyle, 2009; Klotzbach et al., 2018; Pielke et al., 2008; Weinkle et al., 2012; Zeng et al., 2009). Estimating the correlation between storm frequency and the warbler's 19th century distribution and abundance is out of reach because of insufficient data. Evaluating the modern connection is equally challenging because there are relatively few sizeable forest tracts that are untouched by commercial forestry. However, the warbler's well‐documented response to forestry treatments provides compelling evidence of the crucial role that catastrophic storms once played in its breeding ecology. Commercial forestry is a functional analog of tornadoes and hurricanes in its capacity to thin or level extensive tracts of mature forest within short time frames. A comprehensive review of breeding habitat physiognomy suggests that, from the warbler's perspective, there may be little discernible difference between natural and anthropogenic forest disturbance at comparable scales.

The majority of contemporary Swainson's warbler populations are found on actively managed forestry lands (Eddleman et al., 1980; Graves, 2002; Peters et al., 2005; Twedt & Somershoe, 2009). The highest breeding concentrations were recorded in Louisiana (Graves, 2002) and South Carolina (Thompson, 2005), where densities reached 20.5 territories km^−2^ and 17 territories km^−2^, respectively. These figures narrowly exceed the theoretical maximum predicted from average territory size (6.5 ha) (Anich et al., 2009). Warblers colonize large disturbance gaps of anthropogenic or natural origin when regenerating vegetation attains the necessary structural complexity and understory density, typically >35,000 small woody stems ha^−1^ in deciduous woodland (Graves, 2002). The time lag between a forest disturbance event and warbler colonization depends on disturbance intensity, disturbance area, and vegetation growth rates. Large regenerating clearcuts in the Gulf Coast states typically provide suitable breeding habitat for 15–25 years, beginning 4–6 years after harvest (Graves, 2002; Peters et al., 2005; Thompson, 2005). Colonization trajectories in forest tracts leveled by strong tornadoes are likely comparable.

Breeding territories are characterized by understory thickets and vine tangles that provide visual screening for nesting and foraging sites (Graves, 2002; Graves & Tedford, 2016), as well as small semi‐concealed glades for terrestrial dead‐leaf foraging (Graves, 1998, 2002; Meanley, 1970). Warblers abandon territories when successional canopy closure shades out the understory (Graves & Tedford, 2016). Canopy height and floristics, per se, appear to exert little influence on habitat selection (Graves, 2002) as long as essential understory conditions are met. Soil characteristics, however, appear to influence both local and regional occupancy patterns (Graves, 2001, 2002, 2015). Nevertheless, the majority of outwardly suitable habitat tracts remain unoccupied, particularly on the periphery of the historic breeding range. This is a matter of considerable conservation concern.

Was it possible for tornadoes alone to generate enough forest disturbance to maintain breeding populations of Swainson's Warbler in pre‐anthropogenic landscapes? Acknowledging the pitfalls of extrapolating historical storm frequencies from recent NOAA data (National Centers for Environmental Information, 2022a), the median return interval for tornadoes (EF1–EF5) in the warbler counties in the core breeding range in the Gulf Coast states is approximately 5.4 year per 1000 km^2^. The return interval declines to 9.1 years per 1000 km^2^ on the Atlantic coastal plain, 10.2 years per 1000 km^2^ in the Ozark Mountains, and 32.3 years per 1000 km^2^ in the Appalachian Mountains. The observed 6‐fold decline in tornado frequency from the Gulf Coast states to the Appalachians represents the most significant meteorological gradient identified thus far within the circumscribed breeding distribution of the warbler.

Recent advances in remote sensing have made it possible to document tornado tracks in unprecedented detail, overcoming previous challenges in accurately measuring tornado forest damage (Burow et al., 2020; Cannon et al., 2016; Kingfield & de Beurs, 2017; Molthan et al., 2014; Rodríguez & Bech, 2020; Zenoble & Peterson, 2017). Case examples demonstrate the significant impact of exceptional tornadoes on forest structure. The most catastrophic tornado in recent history, in terms of forest destruction, occurred on 24 April 2010 (EF4) with a track length of 242 km that traversed seven counties in Mississippi (Wilkinson & Crosby, 2010). The tornado caused an estimated 16,700 ha of forest damage, with 3440 ha in Yazoo County alone (Wilkinson & Crosby, 2010). Although the probability of an EF1‐EF5 tornado strike at a randomly‐selected site in a given year is exceptionally low, the aggregate impact of hundreds of tornadoes each year within the warbler's core breeding range was likely sufficient to sustain an extensive patchwork of optimal breeding habitat prior to the extensive clearing of old‐growth forests during the 18th and 19th centuries.

The ecological implications of tornado track direction and geometry for disturbance‐dependent birds in forested landscapes have been overlooked by ecologists. The majority of tornadoes in southeastern North America propagate from the west and move in a northeastward direction (Suckling & Ashley, 2006), perpendicular to the south/north flyways of migratory songbirds (Figure 2a). Linear tornado tracks in forested areas may serve as ecological drift fences for disturbance‐dependent birds during migration and particularly during Lévy movements (Humphries et al., 2012; Reynolds, 2018; Viswanathan et al., 1996) in the pre‐mating and postnatal dispersal periods. Linear tornado tracks (with length‐to‐width ratios ranging from 30:1 to 50:1) likely facilitated the discovery of breeding territories and mates for low‐density species in pre‐anthropogenic landscapes, including the historically rare Swainson's warbler and Bachman's warbler. Unlike tornado tracks, other significant sources of forest disturbance such as hurricanes, fire, drought, ice storms, and insect outbreaks tend to leave footprints with more compact shapes.

**FIGURE 5:**
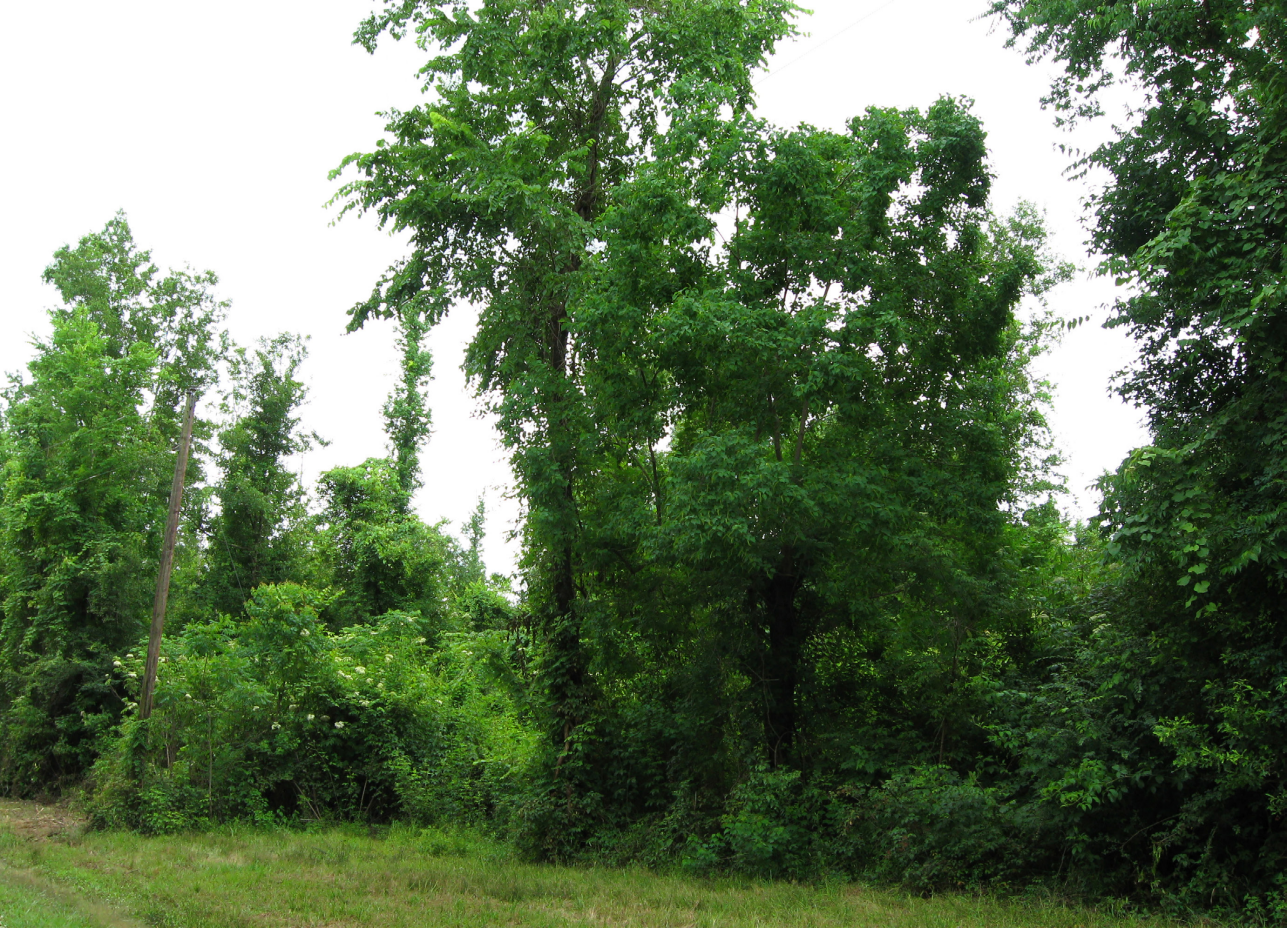
Swainson's warbler territory located in hurricane‐damaged deciduous forest that was characterized by a closed canopy prior to Hurricane Katrina (29 August 2005) and Hurricane Gustav (1 September 2008). Photographed on 25 May 2009 in the Bogue Chitto National Wildlife Refuge, St. Tammany Parish, Louisiana.

The coastal plain from eastern Texas to North Carolina experiences the highest frequency of hurricanes in the Western Hemisphere (Cannon et al., 2023; Doyle, 2009; Weinkle et al., 2012). While hurricanes are less frequent than tornadoes, they cause significantly more damage per event owing to their greater size and longer duration (Peterson, 2000; Zeng et al., 2009). Forest impact models estimate that hurricanes destroyed an average of 147 million trees per year in the United States from 1851 to 1900, and 72 million trees per year from 1900 to 2000 (Zeng et al., 2009). The higher estimates before 1900 can be attributed to increased hurricane activity from 1870 to 1900, before many coastal regions were logged. The detrimental impact of hurricanes on avian species reliant on mature forest, notably the red‐cockaded woodpecker (*Picoides borealis*), has received considerable attention in this region (Lucash et al., 2022). In contrast, the collateral benefits for disturbance‐dependent species have been relatively understudied. Hurricane Katrina (see Figure 5), which made landfall in the Pearl River basin near the Louisiana‐Mississippi state line on 29 August 2005, presented a rare opportunity to directly document the response of Swainson's warbler to hurricane damage on a long‐term study plot (Brown et al., 2011). Following Katrina, its breeding density and that of several other species that prefer dense understory habitat increased significantly.

In closing, a diverse assemblage of breeding birds in eastern North America is associated with disturbance gaps in predominately‐deciduous forests (Askins, 1998; Hunter et al., 2001), but no species appears to be more reliant on catastrophic storm damage than Swainson's warbler. Although most forestland in the warbler's contemporary breeding range in the Gulf Coast states is managed for woody biomass production on short rotation cycles, tornadoes and hurricanes continue to play a critical role in creating forest disturbance gaps of necessary size and frequency to support viable breeding populations of this and other disturbance‐dependent species. There is a pressing need to integrate remote sensing data on tornado and hurricane damage into regional habitat monitoring and management programs, particularly in public forestlands in the lower Mississippi Valley and Gulf coastal plain that are largely off‐limits to commercial forestry.

## AUTHOR CONTRIBUTIONS

**Gary R. Graves:** Conceptualization (lead); data curation (lead); formal analysis (lead); funding acquisition (lead); investigation (lead); methodology (lead); project administration (lead); resources (lead); validation (lead); visualization (lead); writing – original draft (lead); writing – review and editing (lead).

## CONFLICT OF INTEREST STATEMENT

The author declares no competing interests.

## Data Availability

All data generated or analyzed during this study are included in the Supporting Information file and the cited NOAA URLs. The Supporting Information file is deposited in Dryad (doi: 10.5061/dryad.gmsbcc2w8).
